# The prognostic value and immune landscape of a cuproptosis-related lncRNA signature in head and neck squamous cell carcinoma

**DOI:** 10.3389/fgene.2022.942785

**Published:** 2022-07-22

**Authors:** Yao jun Li, Hai yan Li, Quan Zhang, Sheng li Wei

**Affiliations:** ^1^ First Teaching Hospital of Tianjin University of Traditional Chinese Medicine, Tianjin, China; ^2^ Tianjin Huanhu Hospital, Tianjin, China; ^3^ Tianjin Union Medical Center, Tianjin, China

**Keywords:** cuproptosis, long non-coding RNA, head and neck squamous cell carcinoma, prognostic signature, immune landscape

## Abstract

**Background:** Cuproptosis has been recognized as a novel regulatory cell death, which has been confirmed to promote the occurrence and development of tumors. However, whether cuproptosis-related lncRNA has an impact on the prognosis of squamous cell carcinoma of the head and neck (HNSCC) is still unclear.

**Methods:** In total, 501 HNSCC tumor samples and 44 normal were downloaded from the TCGA database. Cuproptosis-related lncRNAs were obtained by co-expressed analysis. We got prognostic lncRNA that was associated with cuproptosis by using univariate Cox regression analysis and LASSO Cox regression. Then we constructed and validated the prognostic signature of HNSCC and analyzed the immune landscape of the signature.

**Results:** The Prognostic Signature is based on 10 cuproptosis-related lncRNAs including AC090587.1, AC004943.2, TTN-AS1, AL162458.1, AC106820.5, AC012313.5, AL132800.1, WDFY3-AS2, CDKN2A-DT, and AL136419.3. The results of overall survival, risk score distribution, and survival status in the low-risk group were better than those in the high-risk group. In addition, all immune checkpoint genes involved were significantly different between the two risk groups (p < 0.05). The risk score was positively correlated with Eosinophils. M0 and M2 phenotype macrophages, mast cells activated, NK cells activated, and negatively related with B cells naive, mast cells resting, plasma cells, CD8T cells, T cells follicular helper, T cells regulatory (Tregs). Consensus clustering was identified in molecular subtypes of HNSC. More high-risk samples concentrated in Cluster1, which had a higher Tumor Immune Dysfunction and Exclusion (TIDE) score and Single Nucleotide Polymorphisms (SNP) alternation than Cluster2.

**Conclusion:** Our study elucidated the correlation between cuproptosis-related lncRNA with prognosis and immune landscape of HNSCC, which may provide references for further research on the exploration of the mechanism and functions of the prognosis for HNSCC.

## Introduction

Head and neck squamous cell carcinoma (HNSCC) is the eighth most common malignancy currently in the world, which contributes to the global cancer burden (Sung et al., 2021). Smoking, alcohol consumption, and HPV infection are recognized risk factors for HNSCC (Jethwa and Khariwala, 2017; Sabatini and Chiocca, 2019; Tumban, 2019). The clinical treatment plan of HNSCC is mainly formulated according to the TNM stage of the tumor. The comprehensive treatment is mainly carried out by surgery, radiotherapy, chemotherapy (Marur and Forastiere, 2016), and immunotherapy (Moskovitz et al., 2018). Over the past 30 years, there have been advances in the treatment of HNSCC, but the 5-year survival rate has not improved (Panvongsa et al., 2022). Therefore, early diagnosis, accurate prognosis assessment, and further effective treatment are urgently needed for HNSCC. A comprehensive understanding of the pathogenesis of HNSCC is required for improving disease outcomes.

The concept of cuproptosis was raised by Tsvetkov et al. in a recent article published in *Science* (Tsvetkov et al., 2022). As a novel regulatory cell death (RCD), cuproptosis is different from known death mechanisms (such as pyroptosis, apoptosis, and ferroptosis), but cooper dependent. Copper is one of the essential trace elements in the human body (Kim et al., 2008), which is also a key component in many essential enzymes (Grubman and White, 2014; Matson Dzebo et al., 2016). In organism, the distribution and homeostasis regulation of cooper is dependent on absorption, transportation, storage, and excretion (Narindrasorasak et al., 2004; Wang et al., 2011; Galler et al., 2020; Shi et al., 2020). Copper balance is critical for cellular metabolism and survival (Ge et al., 2021). Aberrant cooper accumulation may promote the malignant transformation of cells (Gunjan et al., 2017). Copper is involved in three key characteristics of cancer progression: cell proliferation, angiogenesis, and metastasis (Hanahan and Weinberg, 2011; Denoyer et al., 2015). The levels of copper in the serum and tumor tissue of cancer patients increase to support the growth demand for copper (Hanahan and Weinberg, 2011). At present, a number of studies have confirmed that the level of copper in serum and tumor tissues of cancer patients is significantly changed from that of normal people (Basu et al., 2013; Ding et al., 2014; Baltaci et al., 2016; Stepien et al., 2017; Saleh et al., 2020). For head and neck malignancy, there are also some relevant evidence of the correlation between copper levels and the tumors. Garofalo et al. (1980) found a significantly higher copper serum level in advanced stages of epidermoid cancers of the head and neck. Mali et al. (1998) explored the correlation between serum copper levels and response to radiotherapy in patients with HNSCC. Therefore, the study of copper and cuproptosis may provide us to explore HNSC with new means, which is of great significance.

Long non-coding RNAs (lncRNAs) have been confirmed to regulate transcription, epigenetic modifications, translation, and post-translational modifications, and interact directly with signal receptors (Arora et al., 2014; Postepska-Igielska et al., 2015; Grelet et al., 2017; Kleaveland et al., 2018; Schmidt et al., 2020). More and more evidence supported the involvement of lncRNAs in cell differentiation, growth, and the pathogenesis of many diseases including cancers. However, cuproptosis is still rarely reported with lncRNAs. Therefore, it is of great significance to further study the relationship between the cuproptosis and lncRNAs, especially in malignant tumors.

Tumor microenvironment (TME) comprises non-malignant cells, blood vessels, nerves, lymphatic organs, lymph nodes, which are located at the center, edge, or near the tumor lesion (Jin and Jin, 2020). The interaction between TME and tumor cells can determine tumor development and fate (Yuan et al., 2021). Exploring the composition and function of TME is crucial for understanding the development of malignancy (Hinshaw and Shevde, 2019). The immunological role of a few lncRNAs in HNSCC has been confirmed (Li et al., 2020a; Ma et al., 2020). However, a great quantity of immune-related lncRNAs still have not been thoroughly mined. In our study, we attempted to analyze the distribution of cuproptosis-related lncRNAs (Cupr-RLs) in HNSCC from a microscopic perspective and establish the prognostic signature of Cupr-RLs (Cupr-RLPS) in HNSCC patients of TCGA-HNSC cohort. We also investigated the correlation between the prognostic model and tumor immune invasion patterns, immunotherapy responses, and sensitivity to targeted drugs. Our findings may contribute to the prognostic prediction and immunotherapy in HNSCC and provide the personalized treatment to patients.

## Materials and methods

### Data preparation and the cuproptosis-related lncRNAs identification

Using the TCGA data portal, we obtained transcriptome profiling data (FPKM) of HNSC patients. The HNSC data set was annotated and subsequently converted into protein-coding genes and lncRNAs by Perl. The lncRNAs were extracted from the transcriptome profiling set. Clinicopathological information was also downloaded from the TCGA data set. After excluding patients without survival information, we merged the lncRNAs’ expressions with 501 patients’ clinical information. Then, a total of 501 HNSC samples and 44 adjacent samples (non-tumor) corresponding with clinical data were included in the study cohort. Expressions of 19 cuproptosis-related genes (NFE2L2, NLRP3, ATP7B, ATP7A, SLC31A1, FDX1, LIAS, LIPT1, LIPT2, DLD, DLAT, PDHA1, PDHB, MTF1, GLS, CDKN2A, DBT, GCSH, and DLST) were obtained. The cuproptosis-related lncRNAs (Cupr-RLs) were identified by using Pearson correlation analysis (|Pearson R|>0.4, *p* < 0.001) (Schober et al., 2018), and the prognostic Cupr-RLs were determined by Univariate Cox regression analysis. The Wilcoxon rank-sum test was used to analyze the different expression levels of Cupr-RLs between tumor tissues and normal tissues.

### Construction and validation of cuproptosis-related lncRNAs prognostic signature

The entire cohort was randomly divided at a 7:3 ratio into training cohort and validation cohort. The chi-square test was used to determine the differences of demographic and clinicopathological characteristics between the two cohorts. Least absolute shrinkage and selection operator (Lasso)-Cox regression analysis (Friedman et al., 2010) and multivariate Cox regression analysis were applied to construct cuproptosis-related lncRNAs prognostic signature (Cupr-RLPS) for HNSC. The signature risk score formula = $\Sigma_{1}^{n}$ Coe $f_{i}$ ×Exp $r_{i}$ (Coe $f_{i}$ = coefficient, Exp $r_{i}$ = expression value of Cupr-RLs). Patients were then divided into the high-risk and the low-risk groups based on the median risk score cutoff and Kaplan–Meier (K–M) curves was generated with a log-rank test approach to compare overall survival (OS) between the high-risk and the low-risk group. The receiver operating characteristic curves (ROC) (Kamarudin et al., 2017) and the area under the curve (AUC) values were implemented *via* “time ROC” package in R software, for which to evaluate the accuracy sensitivity of the Cupr-RLPS. Subsequently, univariate and multivariate Cox regression analyses were used to identify the predictive efficacy of Cupr-RLPS. GSE65858-GPL10558 cohort from Gene Expression Omnibus (GEO) data sets was used as external validation to verify the robustness and replicability of the signature. A stratified analysis based on the clinicopathological features (age, gender, stages, AJCC grade, AJCC T stage, and N stage) was built. Furthermore, a prognostic nomogram was conducted based on risk score and clinical variables.

### Functional enrichment analysis

Gene Ontology (GO) enrichment analysis was conducted to search for gene functions such as molecular functions (MF), cellular components (CC), and biological processes (BP). The Kyoto Encyclopedia of Genes and Genomes (KEGG) was used to explore potential biological signaling pathways. GO and KEGG analyses were performed by the “clusterProfiler” R package. Gene set enrichment analysis (GSEA) was conducted using the Hallmark, C2 KEGG v.7.4, and C5 GO v.7.4 gene sets (http://www.broadinstitute.org/gsea).

### Assessing the tumor microenvironment landscape of cuproptosis-related lncRNAs prognostic signature

The single-sample gene set enrichment analysis (ssGSEA) (Barbie et al., 2009; Bindea et al., 2013) is a widely used bioinformatics algorithm extensively adopted in cancer-related studies, which was used to evaluate the abundance of immune cells and the related functions or pathways (Shen et al., 2019; Shen et al., 2020; Xiao et al., 2020; Kiessler et al., 2021). The ssGSEA can be implemented *via* “GSVA” R package. By utilizing the CIBERSORT algorithm (Newman et al., 2015; Chen et al., 2018), the difference of immune cell infiltration of HNSC patients between the high-risk group and low-risk group patients was evaluated. CIBERSORT is a web portal (http://cibersortx.stanford.edu/) that provides an estimation of the abundances of member cell types in a population of mixed cells by using gene expression data. The correlation between the immune checkpoint gene expressions and the risk score in the two groups was assessed by Pearson’s test. Spearman’s correlation analysis showed the relationship between immune cell infiltration and the risk score.

### Consensus clustering analysis

To further elucidate the characteristics of Cupr-RLPS in HNSC, the overall samples were separated into different clusters using “ConsensusClusterPlus” R package (Wilkerson and Hayes, 2010). The chi-square test was used to determine the differences of demographic and clinicopathological characteristics between the clusters. The survival analysis was to compare the survival outcomes and the “pheatmap” R package (Zhang et al., 2021) was run to visualize the differential expression of Cupr-RLs and clinicopathological parameters in different clusters.

### Tumor microenvironment characterization, tumor immune dysfunction and exclusion, drug sensitivity, and mutation data

To uncover the correlation between the tumor-infiltrating immune cells in different clusters, diverse algorithms including XCELL, TIMER, QUANTISEQ, MCPcounter, EPIC, CIBERSORT, and CIBERSORT-ABS were implemented. Tumor Immune Dysfunction and Exclusion (TIDE, http://tide.dfci.harvard.edu/) algorithm (Fu et al., 2020) was established for predicting whether CUPR-RLPS could benefit patients in HNSC for immunotherapy. Drug sensitivity of HNSC patients was predicted based on the half-maximal inhibitory concentration (IC50) values of HNSC patients estimated by the “pRRophetic” package (Geeleher et al., 2014). The mutation data containing somatic variants were detected by using the “maftools” R package (Mayakonda et al., 2018), followed by the calculation of TMB.

### Statistical analysis

Statistical analyses and visualization were mainly conducted by R version 4.1.3. The Perl programming language (Version 5.30.0.1) was mainly used for data processing. Student’s t-test and one-way ANOVA were used to calculate differences between two groups or more. The Kaplan–Meier analysis was applied to analyze the OS between two groups of patients. Univariate, Lasso, and multivariate Cox regression analyses were established to evaluate the prognostic significance. Pearson correlation analysis was used to get the correlation of gene expression. ROC and its AUC curve were adopted to estimate the reliability and sensitivity of the prognostic signature. Two-sided *p* < 0.05 was regarded as statistically significant.

## Results

### Identification of prognostic cuproptosis-related lncRNAs in HNSC

**FIGURE 1:**
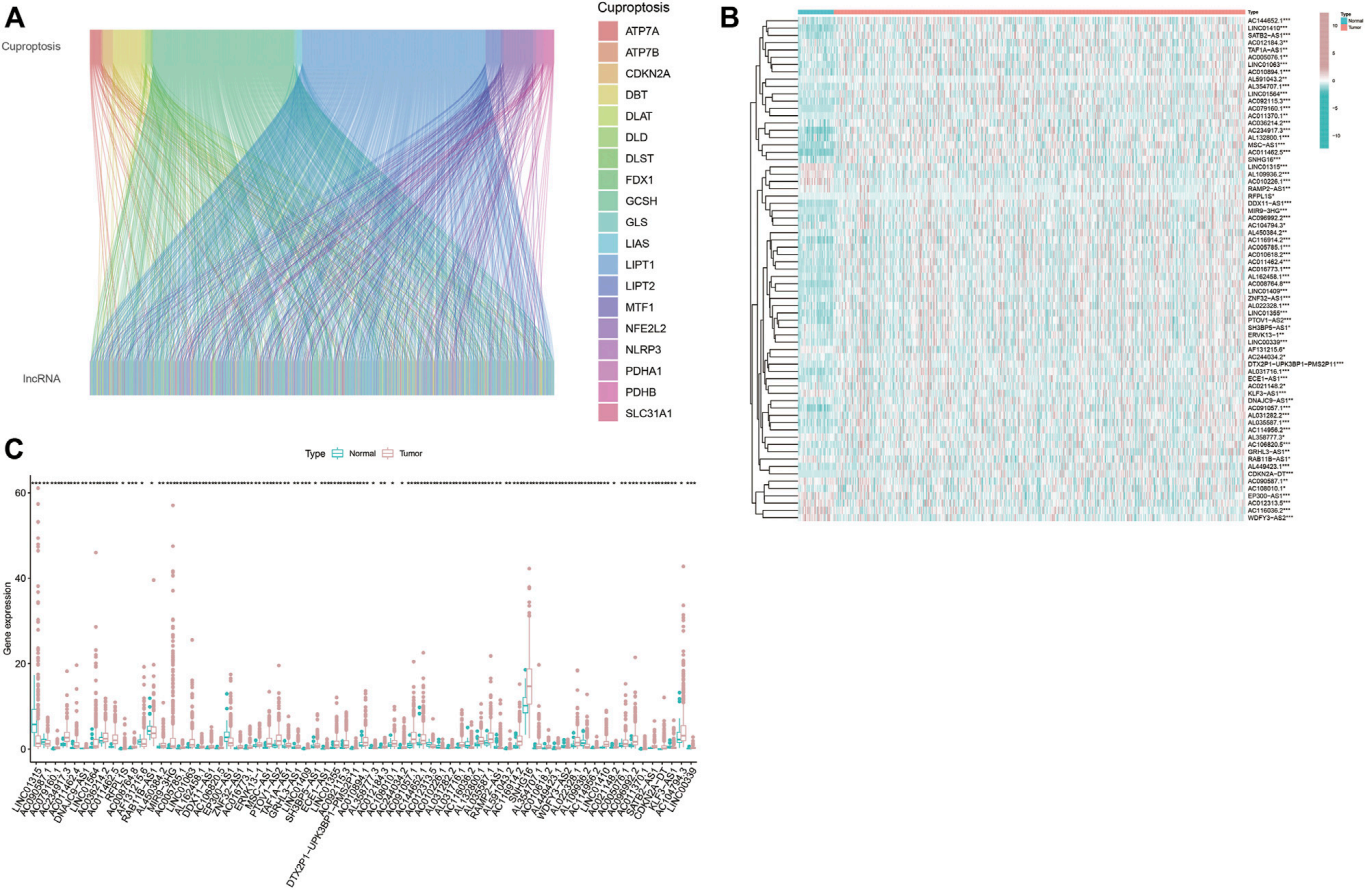
Screen out hub lncRNAs. **(A)** Sankey diagram showed the associations between the 19 cuptoptosis-related genes and the associated lncRNAs. **(B,C)** Differential expressions of the 69 prognostic cuproptosis-related lncRNAs between the normal and tumor samples in HNSC. *p < 0.05, **p < 0.01, ***p < 0.001.

First, 16,876 lncRNAs were screened out from TCGA-HNSC data set, and 783 cuproptosis-related lncRNAs were determined significantly correlated with 19 cuproptosis-related genes by Pearson correlation analysis (Figure 1A). Subsequently, we identified 69 prognostic lncRNAs (Additional file: Supplementary Table S1). The heatmap and box figures show the significant differences in the expression of these 69 OS-related lncRNAs in normal and HNSC tissues (Figure 1B, C). After excluding patients without tumor and survival data, we combined the survival data of 501 patients with cuproptosis-related lncRNA expression data. Detailed clinicopathological information of the patients is given in Table 1.

**TABLE 1:** Demographic and clinicopathological characteristics of patients with HNSCC (*n* = 501).

Characteristics	No.	%
Age (years)		
≤65	326	65.07
>65	175	34.93
Genderact		
Female	133	26.55
Male	368	73.45
Grade		
Grade1	61	12.18
Grade2	299	59.68
Grade3	119	23.75
Grade4	2	0.40
Unknown	20	3.99
Stage		
Stage I	25	4.99
Stage II	69	13.77
Stage III	79	15.77
Stage IV	260	51.90
Unknown	68	13.57
T stage		
T0	1	0.20
T1	45	8.98
T2	133	26.55
T3	96	19.16
T4	171	34.13
Unknown	55	10.98
N stage		
N0	170	33.93
N1	66	13.17
N2	165	32.93
N3	7	1.40
Unknown	93	18.57
M stage		
M0	185	36.92
M1	1	0.20
Unknown	315	62.88

### Construction and validation of the cuproptosis-related lncRNAs prognostic signature

**FIGURE 2:**
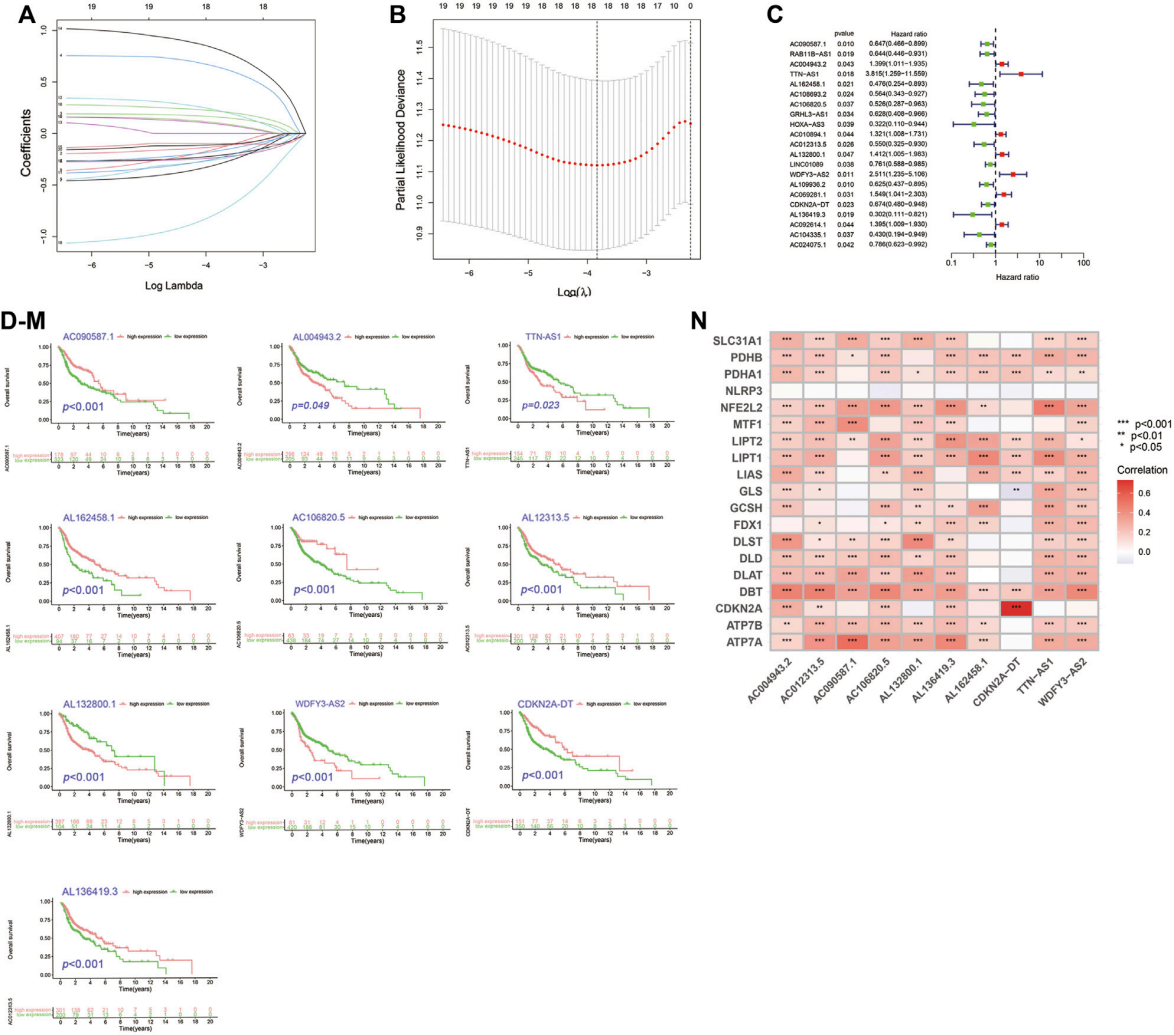
Construction of Cupr-RLs prognostic signature in HNSC. **(A)** The least absolute shrinkage and selection operator (LASSO) Cox regression analysis. **(B)** Lasso coefficient values and vertical dashed lines were calculated at the best log (lambda) value. **(C)** Univariate Cox regression analysis supported the remarkable prognostic significance of the Cupr-RLs. **(D–M)** K–M curves showed different OS with different expression levels of the 10 Cupr-RLs. **(N)** Corrplot displayed the relationship between the 10 Cupr-RLs with 19 cuproptosis-related genes. *p < 0.05, **p < 0.01, ***p < 0.001.

By univariate cox analysis, 69 cuproptosis-related lncRNA (Cupr-RLs) were shown to be significantly associated with the prognosis. In order to avoid the risk of over-fitting, we then used LASSO Cox regression analysis to eliminate the highly correlated ones in the above results and identified a total of 21 Cupr-RLs. The Forest plot showed which were identified as risk factors while hazard ratio (HR)>1, whereas the others were as protective factors (Figures 2A–C). Subsequently, a multivariate Cox regression analysis was used to generate a prognostic signature of 10 Cupr-RLs for OS and the K–M curves showed different OS with different expression levels of the 10 Cupr-RLs (Figures 2D–M). The full names and coefficients of these lncRNAs are shown in Table 2. The signature risk score was used to predict the prognosis of HNSC individuals. Risk score was calculated as follows: Risk score = (-0.3558) × expression of AC090587.1 + 0.3637 x expression of AC004943.2 + 1.4115 x expression of TTN-AS1 + (-0.5844) × expression of AL162458.1 + (-0.6836) × expression of AC106820.5 + (-0.4479) × expression of AC012313.5 + 0.3991 × expression of AL132800.1 + 0.8989 × expression of WDFY3-AS2 + (-0.3509) × expression of CDKN2A-DT + (-0.9749) × Expression of AL136419.3. The corrplot displayed the strong correlation between 10 Cupr-RLs and cuproptosis-related genes (Figure 2N).

**TABLE 2:** The correlation coefficient of Cupr-RLs.

lncRNA	COEF
AC090587.1	−0.3558685458
AC004943.2	0.3633694474
TTN-AS1	1.4115096293
AL162458.1	−0.5844457930
AC106820.5	−0.6835719899
AC012313.5	−0.4479466110
AL132800.1	0.3991427094
WDFY3-AS2	0.8989085686
CDKN2A-DT	−0.3509342783
AL136419.3	−0.9748604128

**FIGURE 3:**
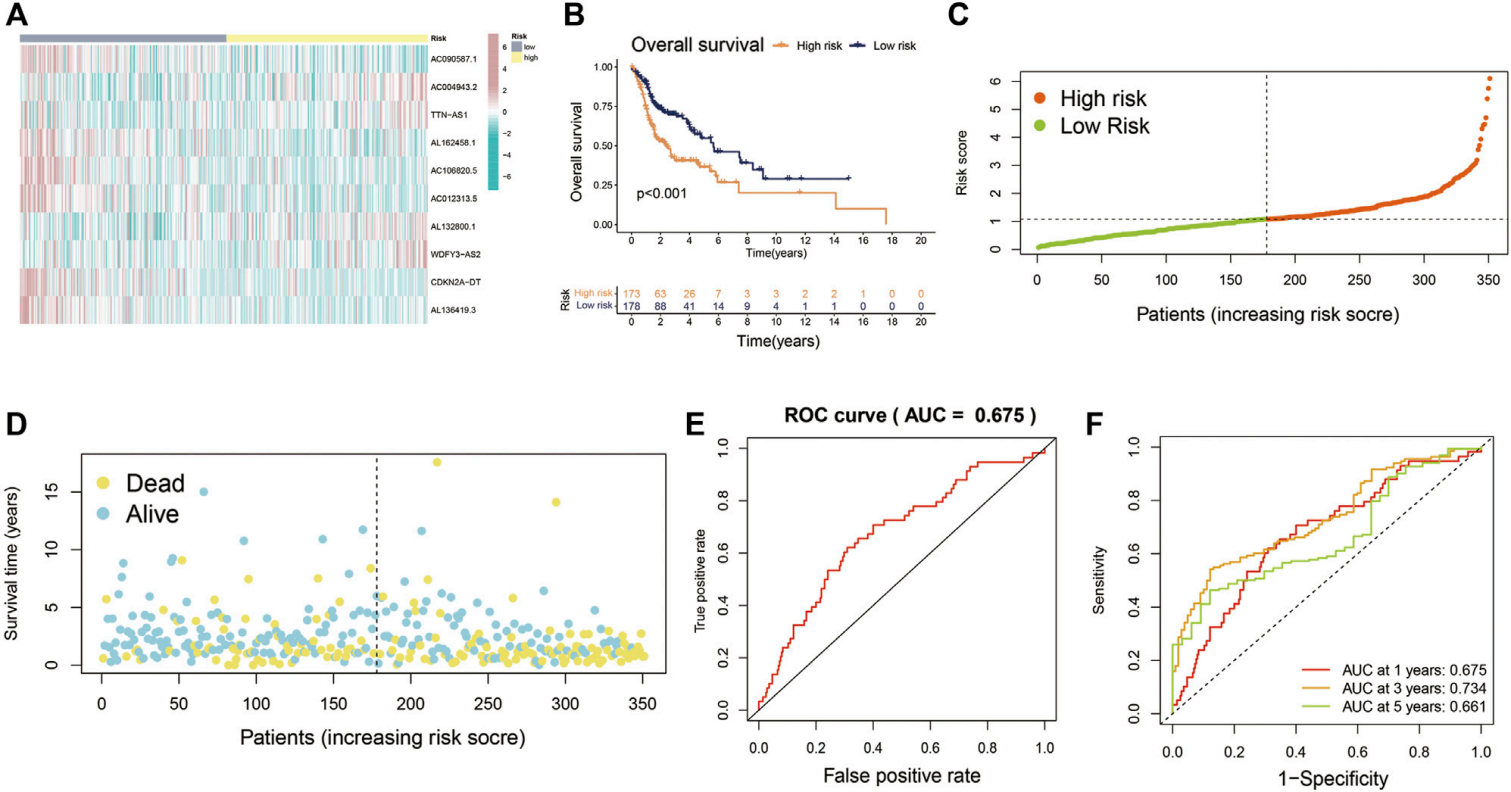
Evaluation of Cupr-RLPS in the training cohort. **(A)** Heatmap showed the expression of the 10 Cupr-RLs in high-risk and low-risk groups in training cohort. **(B)** K-M curves showed the significant differences between the survival outcome in the high-risk group and low-risk group. **(C)** Risk score distribution plot showed the patients’ distribution of HNSC in high-risk and low-risk groups in the training cohort. **(D)** Scatter plot depicted the relationship between the survival status and the risk score in the training cohort of HSNC patients. **(E)** Receiver operating characteristic (ROC) curves represented for the prognostic signature in training cohort. **(F)** AUC value showed 1, 3, and 5 years’ predictions in training cohort.

The entire cohort (*n* = 501) containing complete survival information of patients was randomly divided at a 7:3 ratio into the training cohort (*n* = 351) and the validation cohort (*n* = 150), with no significant differences in terms of any of the clinicopathological parameters between them (Additional file: Supplementary Table S2). Patients were then divided into high-risk and low-risk groups based on the median risk score cutoff. Patients in the training cohort were also put into the two groups by risk scores in order to evaluate the reliability and sensitivity of the Cupr-RLPS. The expression levels distribution of 10 Cupr-RLs in different groups are shown in Figure 3A. OS-time, risk scores distribution plot and scatter plot in the two groups in the training cohort were shown, respectively (Figures 3B–D). According to the description of K–M survival curve, the survival outcome of HNSC patients in the high-risk group was significantly worse than that in the low-risk group. The risk score distribution displayed higher scores in the high-risk group, and the scatter plot indicated that the survival time of HNSC patients in the high-risk group was worse than that in the low-risk group. ROC curves showed the AUC value for Cupr-RLPS was 0.675 (Figure 3E). AUC values corresponding to 1, 3, and 5 years of survival outcomes were 0.675, 0.734, and 0.661 (Figure 3F), suggesting that the Cupr-RLPS harbored a promising prognostic ability in the training group.

**FIGURE 4:**
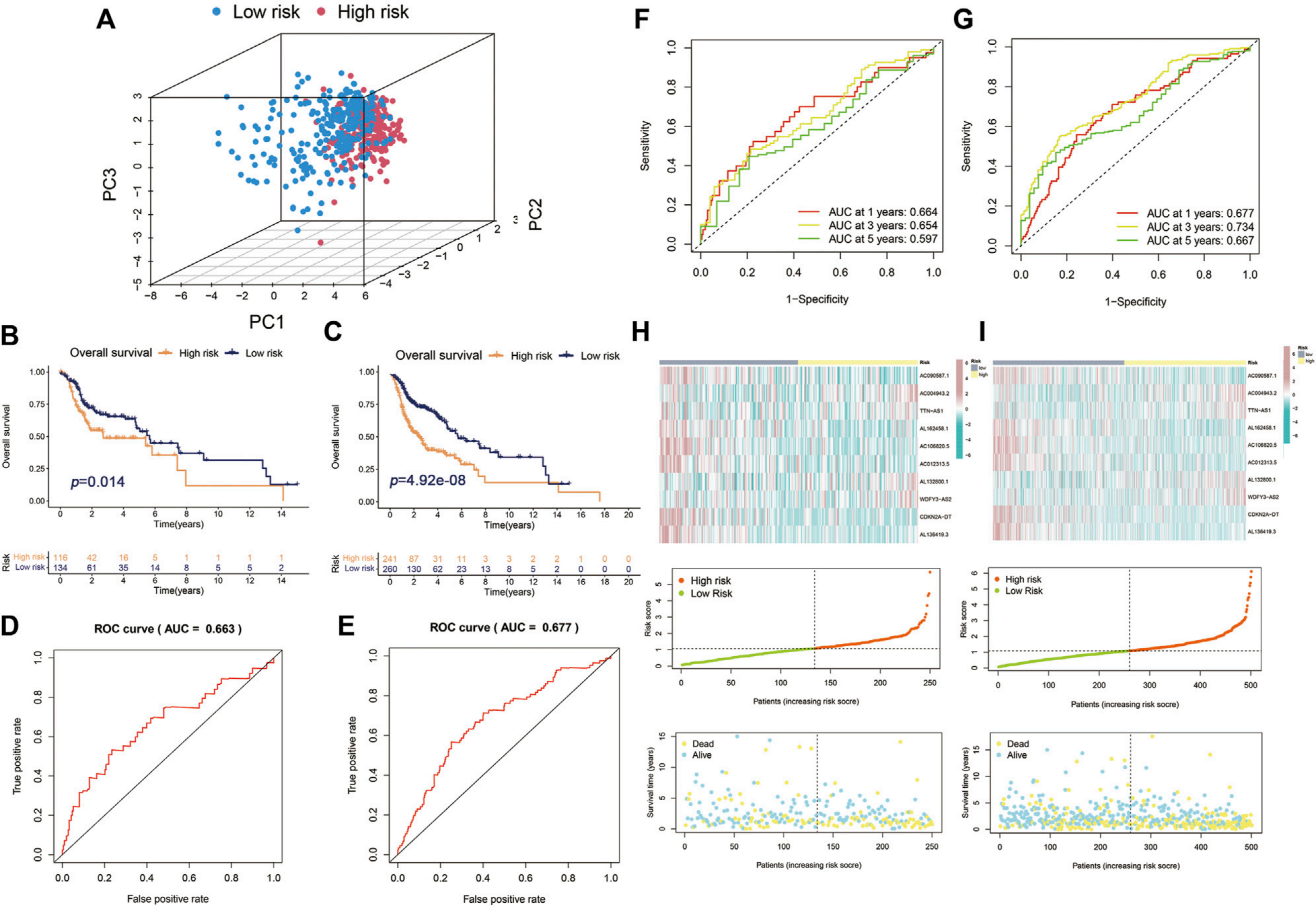
Validation of Cupr-RLPS in testing cohort and overall cohort. **(A)** PCA plots showed the two parts of patients in HNSC separated by risk scores. K–M curves showed the worse OS outcome in the high-risk group in testing cohort **(B)** and overall cohort **(C)**. ROC represented for the prognostic signatures in testing cohort **(D)** and overall cohort **(E)**. AUC values showed 1, 3, and 5 years’ predictions in testing cohort **(F)** and overall cohort **(G)**, respectively. The risk score distribution plot showed the patients’ distribution of HNSC in high-risk and low-risk groups; scatter plot depicted the relationship between the survival status and the risk scores in high-risk and low-risk groups; heatmaps showed the expression of the 10 Cupr-RLs in high-risk and low-risk group in testing cohort **(H)** and overall cohort **(I)**, separately.

Principal components analysis (PCA) analysis revealed that the individuals in high-risk and low-risk groups could be separated into two sections (Figure 4A). Besides, to verify the predictive ability of the Cupr-RLPS to predict prognosis accurately, patients in testing and overall cohorts were also divided into high-risk and low-risk subgroups, and the risk scores were calculated by using the same algorithm as the training cohort. K–M survival displayed that the survival outcomes in testing and overall cohort showed the similar results as the training cohort (Figures 4B,C). ROC curves showed the AUC values were 0.687 and 0.677 in the testing cohort (Figure 4D) and overall cohort (Figure 4E). AUC values corresponding to 1, 3, and 5 years of survival outcomes were 0.687, 0.728, and 0.675 for the testing cohort and 0.677, 0.734, and 0.667 for overall cohort, respectively, which proved the prognostic signature has relatively good value for HNSC patients in testing cohort (Figures 4F,G). The risk score distribution plot and the scatter plot between high risk and low risk in two cohorts depicted the correlations between survival status and risk score of HNSC patients. Two heatmaps showed that the expression profiles of the 10 Cupr-RLs were consistent with those in the training cohort (Figures 4H,I). These results all indicate that the Cupr-RLPS has a stable prognostic-predictive efficiency.

**FIGURE 5:**
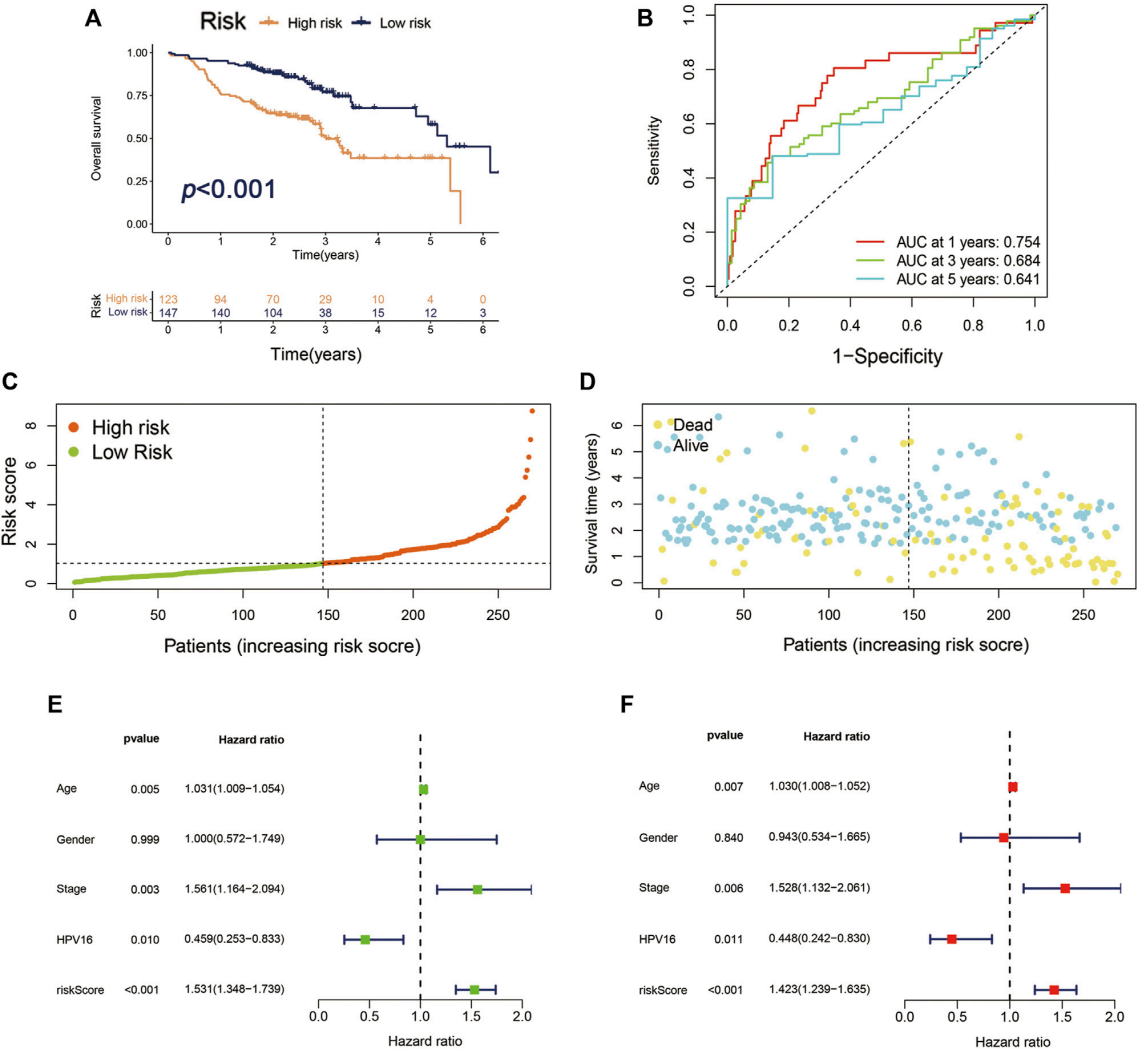
Validation of Cupr-RLPS of GSE65858 cohort. **(A)** K–M curves showed the significant different OS outcomes between the high-risk and low-risk groups. **(B)** AUC values showed 1, 3, and 5 years’ predictions in the cohort. **(C)** The risk score distribution plot displayed the patients’ distribution in high risk and low risk in the cohort. **(D)** The Scatter plot manifested the relationship between the survival status and the risk scores in high-risk and low-risk groups. **(E)** Univariate and **(F)** multivariate Cox regression analysis further indicated that the signature was an independent factor.

To verify the producibility and stability of Cupr-RLPS, the model was applied in the GEO database for external validation. We used the cohort of GSE65858 in the GEO platform as the data sets to verify the classification performance. The main demographic and clinical information are given in Table 3. The risk model was used for calculating the risk score and the samples were divided into high-risk and low-risk groups. Figure 5A shows a significantly different survival outcome between the high-risk group and the low-risk group. AUC values corresponding to 1, 3, and 5 years were 0.754, 0.684, and 0.641 (Figure 5B). Figures 5C,D show the risk score distribution and the survival time of the two groups. The univariate Cox regression analysis (*p* < 0.001) and multivariate Cox regression analysis (*p* < 0.001) indicated the signature was an independent prognostic factor (Figures 5E,F).

**TABLE 3:** Clinicopathological characteristics of HNSC patients in the cohort of GSE65858 in GEO database.

Features	Cohort (*n* = 270)
Age (years)	
≤65	185 (66.67%)
>65	85 (33.33%)
Gender	
Female	47 (17.46%)
Male	223 (82.54%)
Stage	
Stage I	18 (5.82%)
Stage II	37 (13.23%)
Stage III	37 (15.34%)
Stage IV	178 (65.61%)
HPV status	
Positive	73 (24.34%)
Negative	196 (75.13%)
Unknown	1 (0.53%)

We compared the established Cupr-RLPS with 2 other prognostic models: the four-gene signature (Qian et al., 2021) and the eight-lncRNA signature (Lina, 2021) for patients with HNSCC (Supplementary Figure S3; Figure 4). The AUC values for 1, 3, and 5 years survival rates for the four-gene-based model were 0.645, 0.612, and 0.623, which were lower than the AUC values of Cupr-RLPS. Also, the model constructed by eight-lncRNA signatures had worse AUC results than our model. These results showed that our model was better performed for predicting the survival results for HNSCC patients.

### Cuproptosis-related lncRNAs prognostic signature was an independent prognostic indicator in HNSC

**FIGURE 6:**
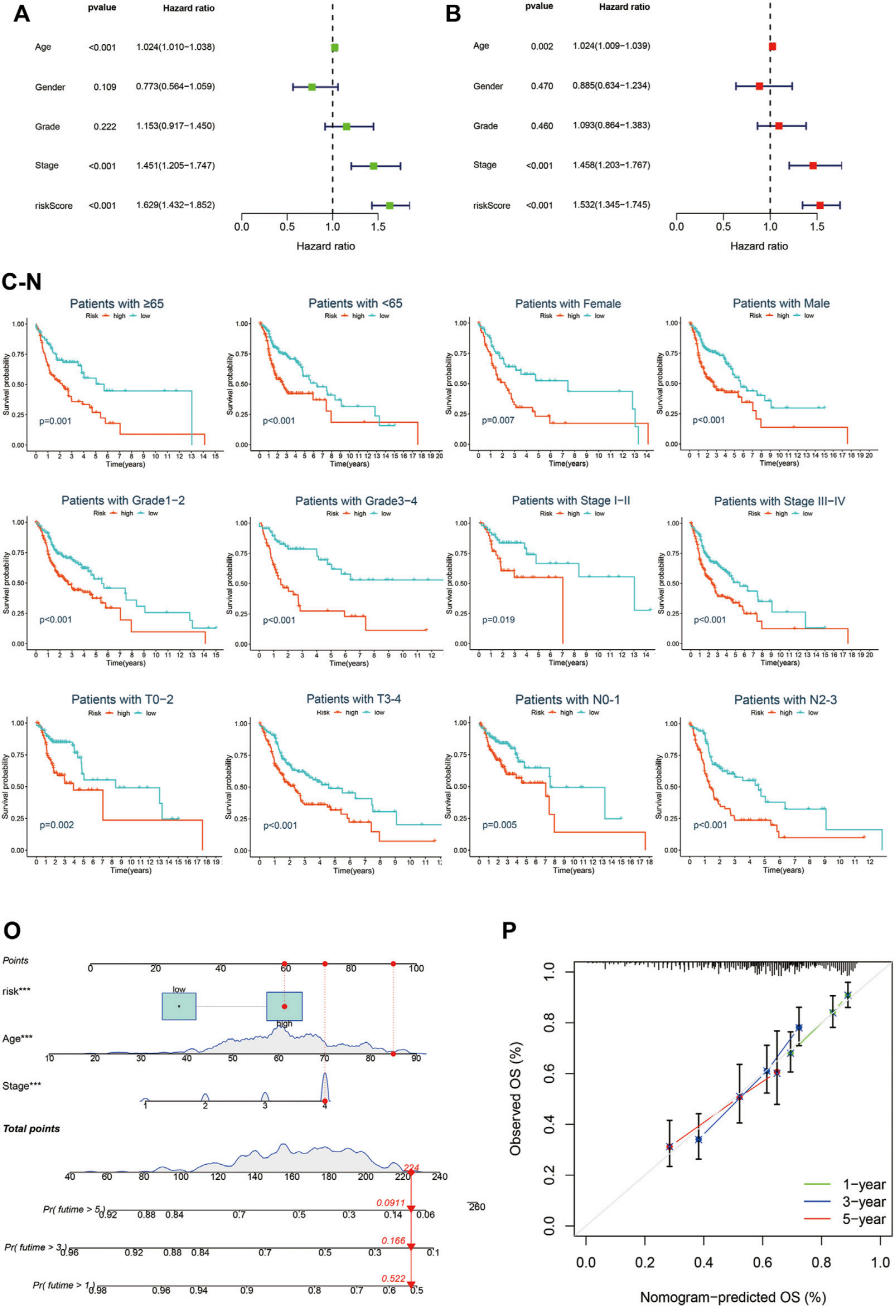
Cupr-RLPS is an independent prognostic indicator. Univariate Cox regression analysis **(A)** and multivariate Cox regression analysis **(B)** all demonstrated the risk score was the independent prognostic factor in the training cohort. Survival analysis stratified by age **(C,D)**, gender **(E,F)**, grade **(G,H)**, clinical-stage **(I,J)**, T stage **(K,L)**, and N stage **(M,N)**. Nomogram predicted the 1, 3, and 5 years’ OS **(O)** and calibration curve **(P)** showed the consistency between the nomogram prediction and the actual survival.

To further identify whether Cupr-RLPS can predicted the prognosis of individuals in HNSC accurately, we conducted univariate and multivariate Cox regression analyses. The results showed that the risk score was the independent prognosis factor of OS in the training cohort (Figures 6A,B). Same conclusions were also validated in the testing cohort (Supplementary Figures S1A,B) and overall cohort (Supplementary Figures S1C,D). Then we performed a stratified analysis based on the clinicopathological features of patients in HNSC to find out whether they were associated with a risk score, so as to explore the clinical application value of the prognostic signature. The clinicopathological features included age, gender, stages, American Joint Committee on Cancer (AJCC) grade, AJCC T stage, N stage. K-M curves were performed and the results showed that in each clinical characteristics, the high-risk group had worse OS than the low-risk group (Figures 6C–N). There we found the significant differences in the clinicopathological features mentioned above between high-risk and low-risk subgroups. Finally, we constructed a Nomogram as an applicable clinical assessment tool to predict the 1, 3, and 5 years’ OS (Figure 6O). The calibration curve showed the consistency between the nomogram prediction and the actual survival (Figure 6P).

### Results of gene ontology, Kyoto encyclopedia of genes and genomes, and gene set enrichment analysis

**FIGURE 7:**
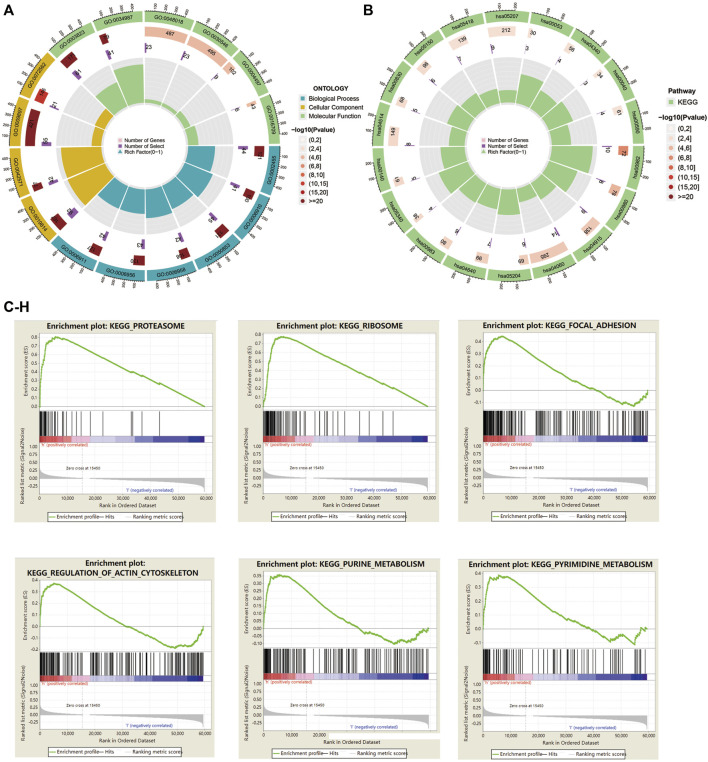
GO, KEGG enrichment, and GSEA analysis in HNSC patients based on the prognostic signature. **(A)** The circle graph showed top GO signaling pathways involved BP, MF, and CC biological processes. **(B)** The circle graph showed top KEGG signaling pathways. **(C–H)** GSEA analysis showed top pathways enriched in the high-risk group.

To elucidate the relationship between the risk model and biological processes, we performed a functional enrichment analysis. DEGs between high-risk and low-risk groups were performed to conduct GO and EKGG enrichment. The top five GO terms for biological processes were humoral immune response mediated by circulating immunoglobulin; phagocytosis, recognition; B cell receptor signaling pathway; complement activation, classical pathway; complement activation. The top four GO terms for cellular components were immunoglobulin complex; immunoglobulin complex, circulating; external side of plasma membrane; blood microparticle. And the top five GO terms for molecular functions were antigen binding; immunoglobulin receptor binding; receptor ligand activity; signaling receptor activator activity; monooxygenase activity (Table 4; Figure 7A). The top five KEGG signaling pathways were drug metabolism-cytochrome P450; metabolism of xenobiotics by cytochrome P450; estrogen signaling pathway; cytokine−cytokine receptor interaction; chemical carcinogenesis-DNA adducts (Table 5; Figure 7B). *Via* GSEA analysis, we selected the top pathways enriched in the high-risk group involving proteasome; ribosome; focal adhesion; regulation of actin cytoskeleton; purine metabolism, and pyrimidine metabolism (Figures 7C–H).

**TABLE 4:** The top 14 GO enrichment terms.

Ontology	ID	Dactescription	*p* Value	Qvalue	Count
BP	GO.0006959	humoral immune response mediated by circulating immunoglobulin	6.43E-43	1.46E-39	44
BP	GO:0006910	phagocytosis, recognition	1.29E-42	1.46E-39	41
BP	GO:0050853	B cell receptor signaling pathway	1.42E-42	1.46E-39	45
BP	GO:0006958	complement activation, classical pathway	1.84E-42	1.46E-39	42
BP	GO:0006956	complement activation	6.89E-40	4.38E-37	43
CC	GO:0019814	immunoglobulin complex	5.08E-112	1.44E-109	92
CC	GO:0042571	immunoglobulin complex, circulating	1.45E-50	2.05E-48	42
CC	GO:0009897	external side of plasma membrane	4.46E-28	4.21E-26	55
CC	GO:0072562	blood microparticle	3.29E-12	2.33E-10	21
MF	GO:0003823	antigen binding	1.04E-60	4.85E-58	61
MF	GO:0034987	immunoglobulin receptor binding	2.82E-49	6.58E-47	41
MF	GO:0048018	receptor ligand activity	0.000103775	0.013869441	23
MF	GO:0030546	signaling receptor activator activity	0.000131995	0.013869441	23
MF	GO:0004497	monooxygenase activity	0.000181884	0.013869441	9

**TABLE 5:** The top 10 KEGG signaling pathways.

ID	Description	*p* Value	Q value	Count
hsa00982	Drug metabolism - cytochrome P450	5.58E-07	0.000119	10
hsa00980	Metabolism of xenobiotics by cytochrome P450	7.52E-05	0.007997	8
hsa04915	Estrogen signaling pathway	0.000847	0.045715	9
hsa04060	Cytokine-cytokine receptor interaction	0.00086	0.045715	14
hsa05204	Chemical carcinogenesis - DNA adducts	0.001471	0.062558	6
hsa04640	Hematopoietic cell lineage	0.002019	0.071563	7
hsa00983	Drug metabolism - other enzymes	0.003136	0.095271	6
hsa05340	Primary immunodeficiency	0.004674	0.111832	4
hsa00140	Steroid hormone biosynthesis	0.004733	0.111832	5
hsa04514	Cell adhesion molecules	0.005458	0.11606	8

### Immune landscapes of cuproptosis-related lncRNAs prognostic signature

**FIGURE 8:**
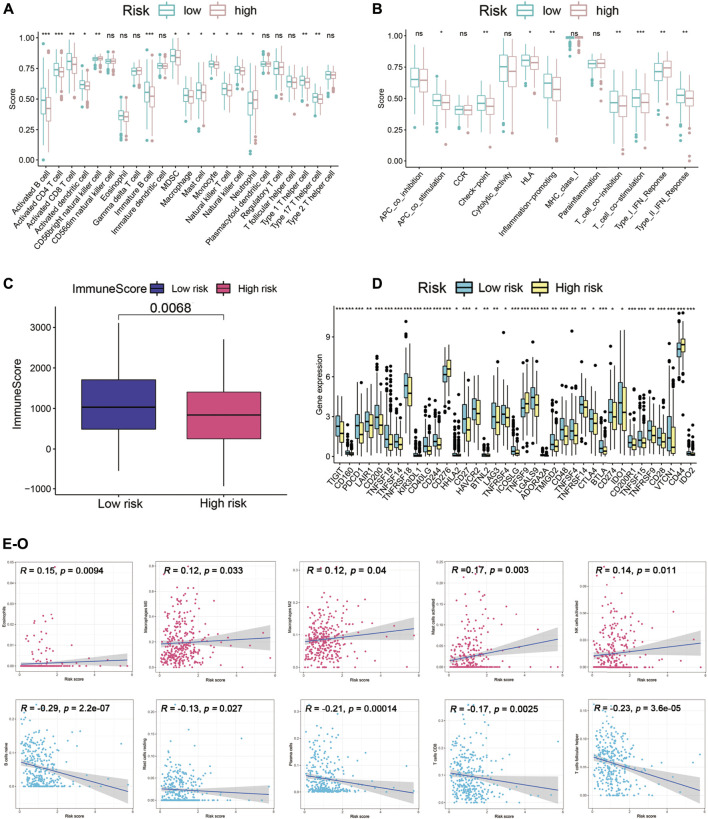
Immune landscape analysis. **(A)** Immune cell scores between high-risk and low-risk groups. **(B)** Immune function scores between high-risk and low-risk groups. **(C)** Different immune scores between two risk subgroups. **(D)** Different expressions of immune checkpoints between high-risk and low-risk groups. **(E–O)** Association between the risk scores and diverse tumor lymph cells. *p < 0.05, **p < 0.01, ***p < 0.001.

To enucleate the immune status correlated with the signature, we assessed the abundance of diverse immune cells and related functions by applying ssGSEA. The immune infiltration of high-risk and low-risk subgroups was evaluated by the CIBERSORT algorithm. Comparison of immune cells and functions confirmed the differences of B cell, CD4T cell, CD8T cell, dendritic cell, CD56 killer cell, immature B cell, immature dendritic cell, MDSC, macrophage, mast cell, monocyte, natural killer T cell, neutrophil, type1T helper cell, type 17 T helper cell, APC co-stimulation, checkpoint, HLA, inflammation promoting, T cell co-inhibition, T cell co-stimulation, type I IFN response, and type II IFN response in high risk and low risk (Figures 8A,B). We also discovered the significant differences of immune checkpoints between two subgroups (Figure 8C). The immune score of the high-risk group was lower than that of the low-risk group (Figure 8D). Besides, scatter plots were generated to show the association between risk score and tumor lymph cells. Spearman’s correlation analysis showed that the immune cell infiltration was positively correlated with Eosinophils, M0 and M2 phenotype macrophages, mast cells activated, NK cells activated, and was negatively related with B cells naïve, mast cells resting, plasma cells, CD8T cell, T cells follicular helper, T cells regulatory (Tregs) (Figures 8E–O). In conclusion, Cupr-RLs of HNSC have a certain correlation with immune cell infiltration.

### Consensus clustering identified in molecular subtypes of HNSC

**FIGURE 9:**
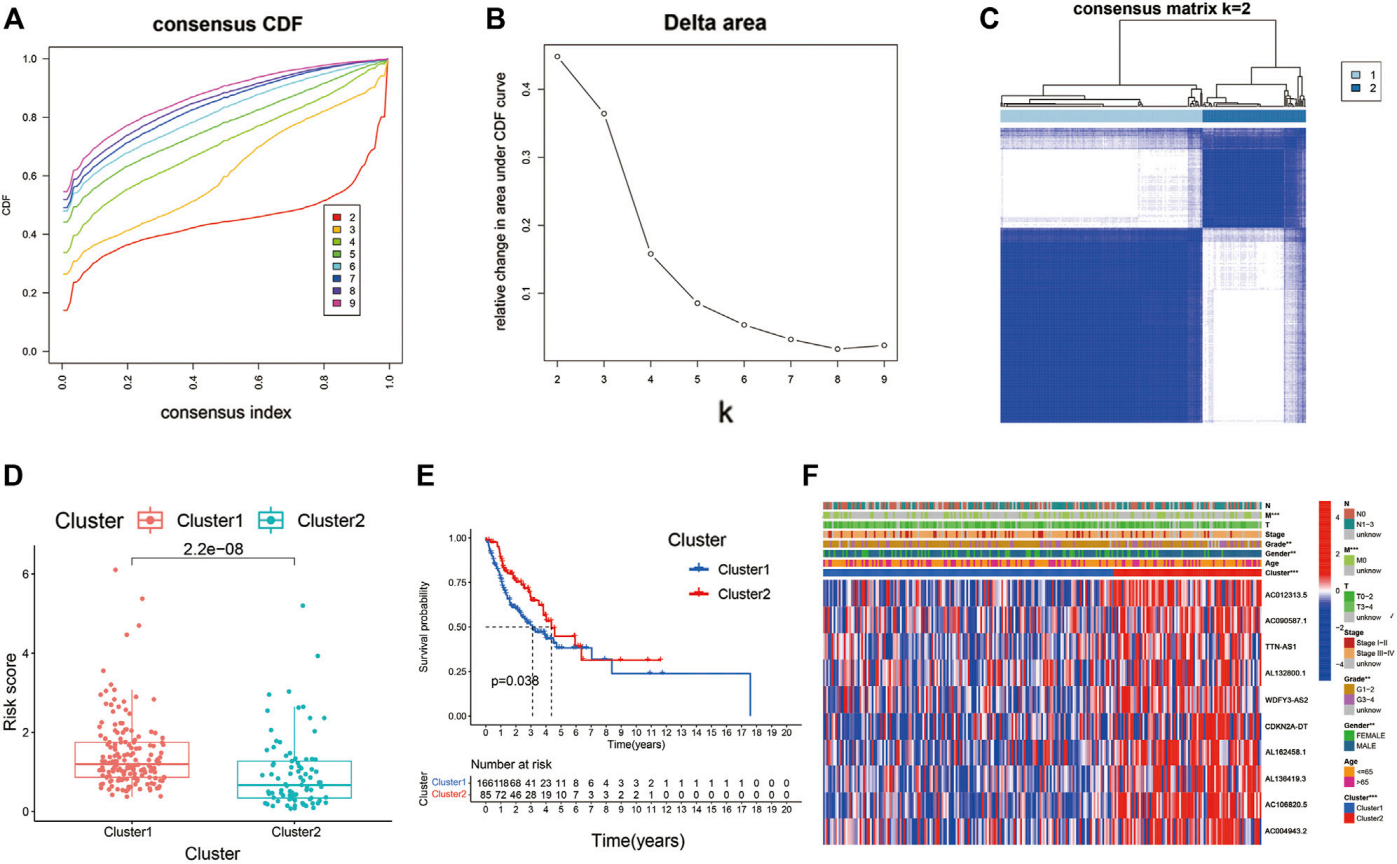
Consensus clustering identified the molecular subtypes of HNSC. **(A)** Consensus clustering cumulative distribution function (CDF) for k = 2 to 9. **(B)** Relative change in area under the cumulative CDF curve for k = 2 to 9. **(C)** Consensus matrix for k = 2. **(D)** The difference of risk scores between Cluster1 and Cluster2. **(E)** The different OS between Cluster1 and Cluster2. **(F)** Relationships between Cupr-RLs expression and clinicopathological parameters. *p < 0.05, **p < 0.01, ***p < 0.001.

Consensus clustering of the Cupr-RLs was used to explore the molecular subtypes of HNSC. To ensure the stability and robustness of the subtype, we filtered the low-expression level of Cupr-RLs and retained which were associated with OS after univariate Cox regression analysis. Then the samples were categorized into two subtypes (Cluster1, *n* = 166; Cluster2, *n* = 85) by consensus clustering while K = 2 (Figures 9A–C). The significant difference of risk scores between Cluster1 and Cluster2 is shown in Figure 9D, which also indicated that the high-risk score sample is mainly concentrated in Cluster1. The K–M curve displayed a survival difference among the two subtypes, of which Cluster1 showed a worse survival outcome compared to Cluster2 (*p* < 0.05) (Figure 9E). Heatmap revealed the relationship between the expression of Cupr-RLs and clinicopathological features parameters (Figure 9F). There were no distinct differences existed in clinical variables between the two clusters.

### GSVA, tumor immune dysfunction and exclusion, drug sensitivity, and SNP alternation of molecular subtypes

**FIGURE 10:**
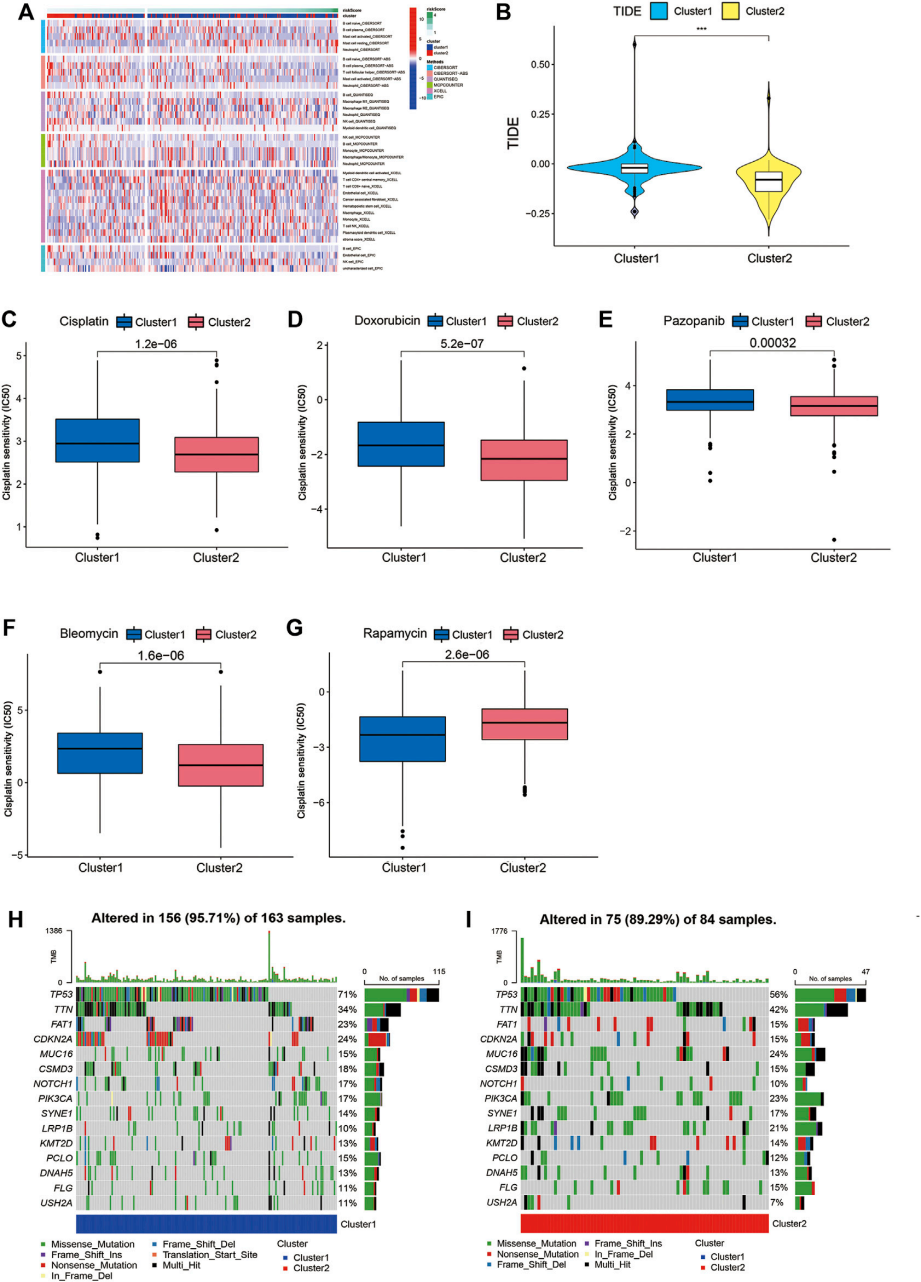
GSVA, TIDE, drug sensitivity and SNP alternation. **(A)** The immune infiltration based on different algorithms. **(B)** TIDE score between Cluster1 and Cluster2. Comparisons of the IC50 values between the two clusters for Cisplatin **(C)**, Doxorubicin **(D)**, Bleomycin **(E)**, Pazopanib **(F)** and Rapamycin **(G)**. **(H,I)** SNP alterations were identified in Cluster1 and Cluster2. *p < 0.05, **p < 0.01, ***p < 0.001.

We used GSVA to seek the associated pathways. Different gene sets between Cluster1 and Cluster2 were displayed. The heatmap showed the immune infiltration based on different algorithms (including TIMER, CIBERSORT, CIBERSORT-ABS, QUANTISEQ, MCPCOUNTER, XCELL, and EPIC) (Figure 10A). TIDE algorithm was established for predicting the immune checkpoint inhibitor (ICI) responders of the two subtypes of patients and further to predict whether Cupr-RLPS could benefit patients in HNSC for immunotherapy. The results showed that Cluster2 responded better than Cluster1 (Figure 10B). In view of the importance of chemotherapeutic agents to HNSC, we selected five commonly used chemotherapeutic drugs and compared the IC50 values between the two subtypes of patients. Our data showed that the IC50 levels of Cisplatin, Doxorubicin, Bleomycin, and Pazopanib (Figures 10C–F) were significantly higher in the Cluster1 than that in Cluster2 and Rapamycin (Figure 10G) was significantly lower in Cluster1 than Cluster2, which were indicated that the HNSC patients in the Cluster1 were more sensitive to these drugs. The correlation between risk score and the IC50 levels of the drugs is shown in Supplementary Figures S2A–E. According to these above results, we came to the conclusion that the consensus clustering has the predictive ability of immunotherapy response and drug sensitivity. In addition, we evaluated the SNP alteration among the two clusters and observed that Cluster1 has a higher SNP alteration than Cluster2 (Figures 10H,I).

## Discussion

As one of the top malignancies in the world, HNSCC is difficult to elucidate the pathogenesis due to its heterogeneity (Raudenska et al., 2021). Therefore, integrating multiple biomarkers into a single model and evaluating its prognostic accuracy as well as its immune relevance and sensitivity to target drugs can individualize the treatment plans and improve the effectiveness.

Copper has been confirmed involved in cancer, but few studies focused on cuproptosis-related lncRNAs. In our present study, we found 69 cuproptosis-related lncRNAs *via* co-expression analysis and after further univariate Cox analysis and LASSO Cox regression, 10 lncRNAs were identified with prognostic values (AC090587.1, AC004943.2, TTN-AS1, AL162458.1, AC106820.5, AC012313.5, AL132800.1, WDFY3-AS2, CDKN2A-DT, AL136419.3). Several evidence showed the dysregulation of TTN-AS1 had pro-oncogenic effects (Zheng et al., 2021a). The aberrant expression of TTN-AS1 played a key role of regulatory in the carcinogenesis of lung cancer, liver cancer, glioblastoma, and breast cancer (Wang et al., 2020a; Fang et al., 2020; Qi and Li, 2020; Tang et al., 2020). AC004943.2 and AC106820.5 were related to HNSCC. As a lncRNA related to the ceRNA network, AC004943.2 showed a value of revealing potential biomarkers in laryngeal squamous cell carcinoma (Jing et al., 2020). AC106820.5 is one of the m6A/m5C/m1A-related lncRNAs that have been constructed to the accuracy prognosis in HNSCC (Wang et al., 2021a). AC012313.5 associated with ferroptosis predicts prognosis in colorectal cancer (Li et al., 2022). WDFY3-AS2 has also been reported correlated with diverse of cancers such as ovarian cancer (Li et al., 2020b), glioma (Zheng et al., 2021b), and esophageal (Kong et al., 2021). The following genes are lack for documented (AC090587.1, AL162458.1, AL132800.1, CDKN2A-DT, and AL136419.3). Thus, more studies are needed to mine the effects of these lncRNAs.

According to the expression level of these 10 Cupr-RLs, we conducted a risk score for each tumor sample to construct a prognostic signature. Through the relationship between risk score, survival status, and survival outcomes, the training and validation set of randomly separated tumor samples proved that the prognostic model composed of 10 lncRNAs could well predict the prognosis of HNSC tumor patients. The univariate and multivariate cox regression analysis indicated the signature could be a prognostic marker independent of age, sex, tumor grade, and stage, which further indicated that the Cupr-RLPS had a certain prognostic value. The GEO cohort used as external validation also proved the robustness and the replicability of the signature. Interestingly, we included the HPV status into the variables in the GEO cohort, the result showed it was not an independent prognostic factor and the specific reasons need our further study. Besides, a newly developed nomogram was expected to improve the clinical decision-making and may guide the development of treatment strategies. By comparison, our signature had a stronger prediction performance of 1, 3 and 5 years than other signatures. Then we explored the correlation between Cupr-RLPS and the immune status. Immune checkpoint inhibitors do not respond well to HNSCC and only a small number of patients benefit from immunotherapy, suggesting that cancer stem cells may have developed other mechanisms to evade immune surveillance. Therefore, to mine the new biomarkers for optimizing treatment strategies is becoming increasingly important. We found that the high expression levels of the immune checkpoint genes in the high-risk group were CD276, TNFRSF9, and CD44. CD276 was highly expressed on the surface of HNSCC and acted as an immune checkpoint to enable cancer stem cells to evade the surveillance of the immune system, while blocking CD276 can effectively enhance T-cell-mediated anti-tumor immunity (Wang et al., 2021b). Recently, a published study showed that TNFRSF9 agonist combined with PD-L1 could effectively activate and amplify tumor-specific cytotoxic T cells, enhancing tumor control and killing (Geuijen et al., 2021). CD44 reduced the sensitivity of tumor cells to CTL by down-regulating the Fas-FasL pathway, leading to tumor escape from CTL killing (Wang et al., 2020b). TME acted a great role in tumorigenesis and progression of HNSCC (Wijetunga et al., 2021). We analyzed the association between the Cupr-RLs and TME immune activity and found the correlation between them. Among tumor infiltration cells, the levels of Eosinophils, M0 and M2 phenotype macrophages, mast cells activated, NK cells activated were positively correlated with the risk score in high-risk and low-risk groups. There were evidence that the TICs mentioned above were related with tumorigenesis, tumor progression, and immunotherapy efficacy in HNSCC (Nishikawa et al., 2021; Chen et al., 2021; Jin and Qin, 2020; Moreno-Nieves et al., 2021). In addition, tumor samples were divided into Cluster1 and Cluster2 according to the lncRNAs expression levels by using consensus clustering analysis. Cluster1 showed a higher risk score and a worse survival outcome compared to Cluster2, which was consistent with the fact that more high-risk samples were concentrated in Cluster1. TIDE score indicated that Cluster1 had a worse response to chemotherapeutics than Cluster2. Therefore, the prognostic signature had the potential predictive ability of HNSCC and may provide references for further research on the exploration of the mechanism and function of the prognostic value. In summary, the study successfully constructed and verified the prognostic risk model of cuproptosis-related lncRNA based on TCGA and GEO databases and also analyzed the immune landscape associated with the model in HNSCC. The study had a few limitations. First, due to different gene sequencing in different databases, not all lncRNAs were included in the study for analysis. Second, more samples and risk factors need to be recruited to build a more accurate model. In addition, the study preliminarily explored the molecular mechanism of cuproptosis-related lncRNA in HNSCC, and the specific functional mechanism is worthy of further study.

## Conclusion

In conclusion, we identified 10 Cupr-RLPS with potential prognostic value in HNSCC and a prognostic and predictive Cupr-RLPS was developed. It is helpful to study the molecular mechanism of HNSC tumorigenesis and predict the therapeutic effect of HNSCC patients.

## Data Availability

The data sets presented in this study can be found in online repositories. The names of the repository/repositories and accession number(s) can be found in the article/Supplementary Material
