# Supplementary material for: The prognostic value and immune landscape of a cuproptosis-related lncRNA signature in head and neck squamous cell carcinoma
Source: Front Genet. 2022 Jul 22;13:942785. doi: 10.3389/fgene.2022.942785 (PMC9356288; doi:10.3389/fgene.2022.942785)
Supplement: Supplementary file 1 [file Presentation1.zip › Figure 1.DOCX]

| **cuproptosis-related lncRNA**  **Table S1. The 69 cuproptosis-related prognostic lncRNAs.** | **HR** | **HR.95L** | **HR.95H** | **P-value** |
| --- | --- | --- | --- | --- |
| **LINC01315**  **AC090587.1**  **AC079160.1**  **AC234917.3**  **AC011462.4**  **DNAJC9-AS1**  **LINC01564**  **AC036214.2**  **AC011462.5**  **RFPL1S**  **AC008764.8**  **AF131215.6**  **RAB11B-AS1**  **AL450384.2**  **MIR9-3HG**  **AC005785.1**  **LINC01063**  **AL162458.1**  **DDX11-AS1**  **AC106820.5**  **EP300-AS1**  **ZNF32-AS1**  **AC016773.1**  **ERVK13-1**  **MSC-AS1**  **PTOV1-AS2**  **TAF1A-AS1**  **GRHL3-AS1**  **LINC01409**  **SH3BP5-AS1**  **ECE1-AS1**  **LINC01355**  **AC092115.3**  **DTX2P1-UPK3BP1-PMS2P11**  **AC010894.1**  **AL358777.3**  **AC012184.3**  **AC108010.1**  **AC244034.2**  **AC091057.1**  **AC144652.1**  **AC012313.5**  **AC010226.1**  **AL031282.2**  **AL031716.1**  **AC116036.2**  **AL132800.1**  **AL035587.1**  **RAMP2-AS1**  **AL591043.2**  **AC116914.2**  **SNHG16**  **AL354707.1**  **AC010618.2**  **AL449423.1**  **WDFY3-AS2**  **AL022328.1**  **AL109936.2**  **AC114956.2**  **LINC01410**  **AC021148.2**  **AC005076.1**  **AC096992.2**  **AC011370.1**  **SATB2-AS1**  **CDKN2A-DT**  **KLF3-AS1**  **AC104794.3**  **LINC00339** | 0.9669  0.8361  1.3125  1.0900  0.8494  0.4060  1.0301  1.0877  1.0875  0.6500  0.6682  0.8903  0.8888  0.8291  0.9629  0.5959  1.0518  0.6250  0.7857  0.5638  0.8976  0.7587  0.7174  0.8296  1.0906  0.9022  1.1637  0.6789  0.2800  0.7831  0.3705  0.8685  1.1227  0.3312  1.1125  0.6102  1.5261  0.8781  0.7689  0.9307  1.0537  0.7871  0.3053  0.4110  0.8580  1.2480  1.1170  0.9364  0.8318  1.7619  0.9026  1.0229  1.0848  0.7599  0.6352  1.8236  0.9074  0.8032  0.5895  1.1369  0.4191  1.0924  0.9270  1.4448  1.2275  0.7383  0.6883  0.9526  0.4875 | 0.9420  0.7343  1.0504  1.0161  0.7441  0.1757  1.0020  1.0164  1.0103  0.4492  0.4716  0.8201  0.8265  0.7036  0.9369  0.3689  1.0041  0.4309  0.6185  0.3690  0.8300  0.5883  0.5351  0.6896  1.0200  0.8375  1.0081  0.5345  0.0862  0.6489  0.1824  0.7680  1.0462  0.1364  1.0402  0.3781  1.1180  0.7794  0.6142  0.8722  1.0020  0.6224  0.1348  0.2381  0.7443  1.0312  1.0201  0.8781  0.7022  1.0470  0.8328  1.0031  1.0062  0.5902  0.4454  1.1632  0.8306  0.6890  0.3476  1.0292  0.2393  1.0135  0.8633  1.0952  1.0284  0.6193  0.4983  0.9218  0.2719 | 0.9924  0.9522  1.6400  1.1694  0.9695  0.9377  1.0590  1.1639  1.1706  0.9405  0.9466  0.9665  0.9557  0.9770  0.9897  0.9627  1.1017  0.9064  0.9981  0.8614  0.9707  0.9785  0.9618  0.9981  1.1661  0.9718  1.3433  0.8623  0.9096  0.9451  0.7526  0.9822  1.2048  0.8038  1.1898  0.9848  2.0833  0.9894  0.9626  0.9930  1.1081  0.9955  0.6915  0.7095  0.9889  1.5104  1.2231  0.9984  0.9853  2.9652  0.9782  1.0431  1.1695  0.9784  0.9060  2.8591  0.9913  0.9364  0.9998  1.2558  0.7341  1.1775  0.9953  1.9059  1.4652  0.8802  0.9508  0.9844  0.8740 | 0.0114  0.0069  0.0167  0.0162  0.0156  0.0348  0.0353  0.0151  0.0255  0.0223  0.0233  0.0055  0.0015  0.0253  0.0070  0.0344  0.0331  0.0132  0.0482  0.0081  0.0068  0.0334  0.0264  0.0477  0.0111  0.0067  0.0385  0.0015  0.0342  0.0108  0.0060  0.0247  0.0013  0.0146  0.0019  0.0431  0.0078  0.0327  0.0219  0.0299  0.0416  0.0458  0.0044  0.0014  0.0346  0.0229  0.0169  0.0446  0.0331  0.0329  0.0125  0.0230  0.0339  0.0332  0.0122  0.0088  0.0313  0.0051  0.0499  0.0115  0.0024  0.0209  0.0367  0.0092  0.0232  0.0007  0.0234  0.0037  0.0159 |

**HR, hazard ratio**
