# Supplementary material for: The prognostic value and immune landscape of a cuproptosis-related lncRNA signature in head and neck squamous cell carcinoma
Source: Front Genet. 2022 Jul 22;13:942785. doi: 10.3389/fgene.2022.942785 (PMC9356288; doi:10.3389/fgene.2022.942785)
Supplement: Supplementary file 1 [file Presentation1.zip › Figure 2.DOCX]

**Table S2. Clinicopathological characteristics of HNSC patients in the training and testing cohorts in TCGA.**

| **Features** | **Training cohort(n=351)** | **Testing cohort(n=150)** | **P-value** |
| --- | --- | --- | --- |
| **Age(years)** |  |  | 0.855 |
| ≤65 | 227(64.67%) | 99(66.00%) |  |
| >65 | 124(35.33%) | 51(34.00%) |  |
| **Gender** |  |  | 0.711 |
| Female | 91(25.93%) | 42(28.00%) |  |
| Male | 260(74.07%) | 108(72.00%) |  |
| **Grade** |  |  | 0.417 |
| Grade1  Grade2 | 39(11.11%)  218(62.11%) | 22(14.67%)  81(54.00%) |  |
| Grade3  Grade4 | 81(23.08%)  1(0.28%) | 38(25.33%)  1(0.67%) |  |
| Unknown | 12(3.42%) | 8(5.33%) |  |
| **Stage** |  |  | 0.339 |
| Stage I  Stage II | 15(4.27%)  45(12.82%) | 10(6.67%)  24(16.00%) |  |
| Stage III  Stage IV | 58(16.52%)  190(54.13%) | 21(14.00%)  70(46.67%) |  |
| Unknown | 43(12.25%) | 25(16.67%) |  |
| **T stage** |  |  | 0.182 |
| T0-  T1 | 1(0.28%)  28(7.98%) | 0(0%)  17(11.33%) |  |
| T2  T3  T4 | 88(25.07%)  74(21.08%)  127(36.18%) | 45(30.00%)  22(14.67%)  44(29.33%) |  |
| Unknown | 33(9.40%) | 22(14.67%) |  |
| **N stage** |  |  | 0.885 |
| N0  N1  N2 | 126(35.90%)  46(13.11%)  117(33.33%) | 44(29.33%)  20(13.33%)  48(32.00%) |  |
| N3 | 5(1.42%) | 2(1.33%) |  |
| Unknown | 57(16.24%) | 36(24.00%) |  |
