# Supplementary material for: The prognostic value and immune landscape of a cuproptosis-related lncRNA signature in head and neck squamous cell carcinoma
Source: Front Genet. 2022 Jul 22;13:942785. doi: 10.3389/fgene.2022.942785 (PMC9356288; doi:10.3389/fgene.2022.942785)
Supplement: Supplementary file 1 [file Presentation1.zip › Figure 3.DOCX]

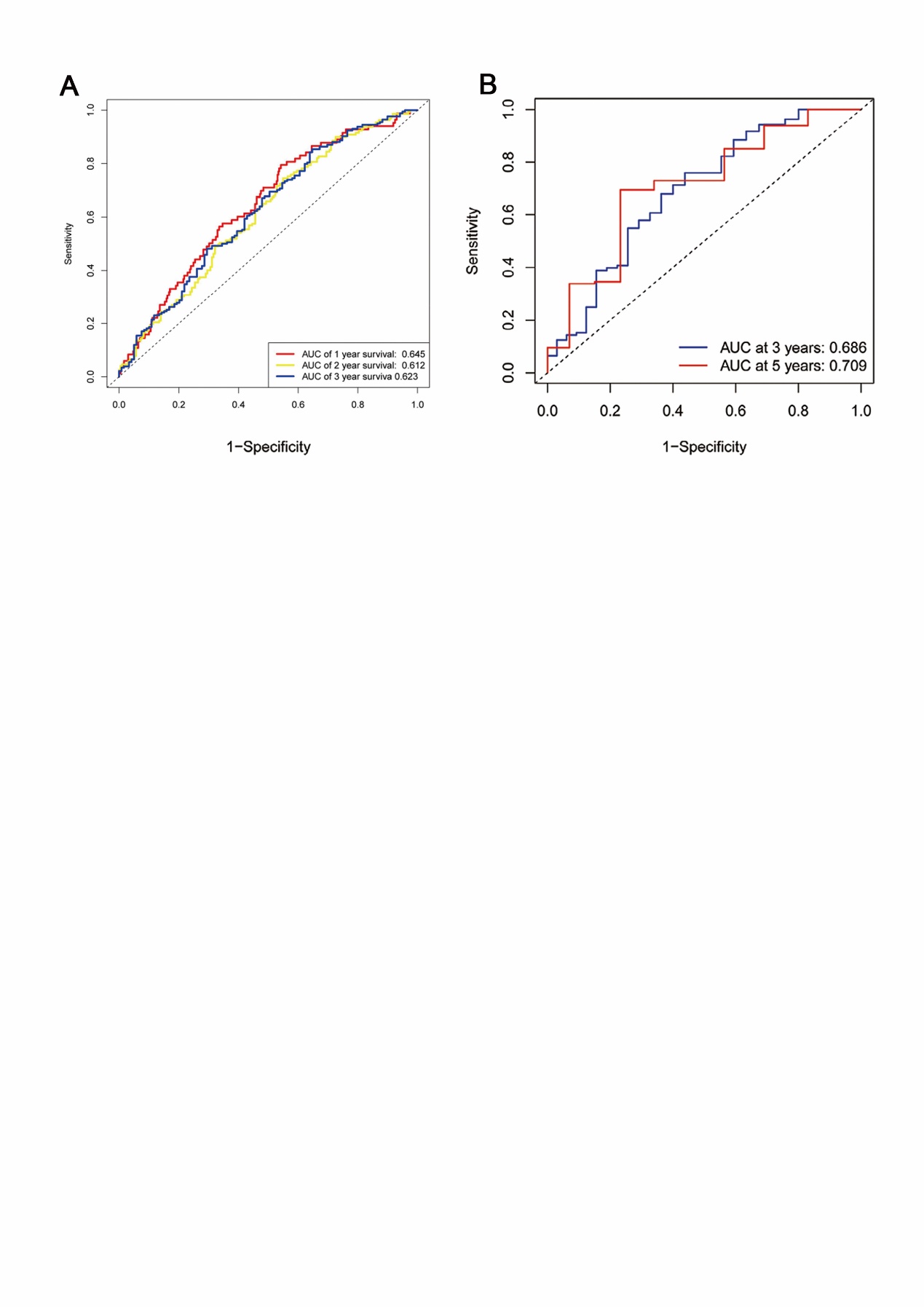


Figure S3.

1. AUC values showed 1-, 3-, and 5-years’ predictions in the four-gene-signature risk model.
2. AUC values showed 3-, and 5-years’ predictions in the eight-lncRNA-signature risk model.
